# From haystack to high precision: advanced sequencing methods to unraveling circulating tumor DNA mutations

**DOI:** 10.3389/fmolb.2024.1423470

**Published:** 2024-08-06

**Authors:** Tamires Ferreira da Silva, Juscelino Carvalho de Azevedo, Eliel Barbosa Teixeira, Samir Mansour Moraes Casseb, Fabiano Cordeiro Moreira, Paulo Pimentel de Assumpção, Sidney Emanuel Batista dos Santos, Danielle Queiroz Calcagno

**Affiliations:** ^1^ Programa de Residência Multiprofissional em Saúde (Oncologia), Hospital Universitário João de Barros Barreto, Universidade Federal do Pará, Belém, Brazil; ^2^ Núcleo de Pesquisas em Oncologia, Universidade Federal do Pará, Belém, Brazil

**Keywords:** precision medicine, ctDNA mutation, non-targeted next-generation sequencing, targeted next-generation sequencing, bioinformatics

## Abstract

Identifying mutations in cancer-associated genes to guide patient treatments is essential for precision medicine. Circulating tumor DNA (ctDNA) offers valuable insights for early cancer detection, treatment assessment, and surveillance. However, a key issue in ctDNA analysis from the bloodstream is the choice of a technique with adequate sensitivity to identify low frequent molecular changes. Next-generation sequencing (NGS) technology, evolving from parallel to long-read capabilities, enhances ctDNA mutation analysis. In the present review, we describe different NGS approaches for identifying ctDNA mutation, discussing challenges to standardized methodologies, cost, specificity, clinical context, and bioinformatics expertise for optimal NGS application.

## Background

Cancer is a multifaceted and constantly evolving disease, which has a progression of genetically distinct clones that guide its course (Lomakin et al., 2022). In the era of precision medicine, the identification of mutations within cancer-associated genes assumes paramount significance, as it serves as a compass guiding the therapeutic journey for patients (Malone et al., 2020).

As a groundbreaking stride, liquid biopsies have risen as a complementary approach to traditional tissue biopsies, offering molecular insights into tumors that can revolutionize early cancer detection, patient stratification, treatment efficacy assessment, and post-treatment vigilance. Unlike tissue biopsies, this minimally invasive approach stands out for its increased uniformity, mitigating sampling bias across diverse tumor regions (Martins et al., 2021). Central to this methodology are mainly circulating tumor DNA (ctDNA) and circulating tumor cells (CTCs) (Jiang et al., 2021).

In particular, ctDNA corresponds to DNA fragments at about 160–200 base pairs (bp) that contain tumor-specific mutations which potentially represent the real-time status of the tumor genome (Chen and Zhao, 2019; Noguchi et al., 2020; Yu et al., 2022). Consequently, the assessment of ctDNA at specific time points—such as the clinical management and the detection of minimal residual disease (MRD)—has emerged as a pivotal factor in prognostication for a multitude of cancer types, encompassing breast cancer, colorectal cancer and leukemia (Parikh et al., 2021; Fürstenau et al., 2022; Turner et al., 2023).

**FIGURE 1:**
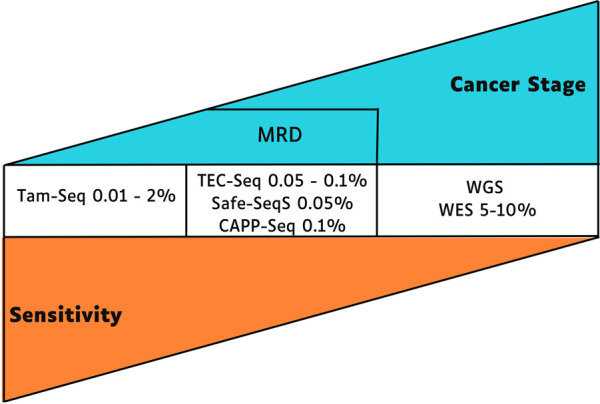
Sensitivity and applicability of techniques for identifying ctDNA mutations, the early stage of cancer requires more sensitive next-generation sequencing techniques to detect mutations in ctDNA. MRD, minimal residual disease; TEC-Seq, Targeted error correction sequencing; Safe-SeqS, Safe-Sequencing System; CAPP-Seq, Cancer Personalized Profiling by Deep Sequencing; WGS, Whole-Genome Sequencing; WES, Whole Exome Sequencing.

The ctDNA concentrations represent about 0.01% of cell-free DNA (cfDNA); these low percentages lead to challenges in acquiring enough quality material for detection, especially at the early stages of tumor development (Huerta et al., 2021). According to individual tumor features, a specific analysis methodology is required, and the technique’s sensitivity for identifying ctDNA mutations is inversely proportional to the tumor stage (Elazezy and Joosse, 2018; Oliveira et al., 2020; Sanz-Garcia et al., 2022) (Figure 1).

In 2016, the U.S. Food and Drug Administration (FDA) and the European Medicines Agency approved the first ctDNA-based test to prescribe *EGFR* inhibitors in patients with non-small cell lung cancer (NSCLC) - Cobas *EGFR* mutation test v2 (Kwapisz, 2017; U.S Food and Drug Administration, 2022; U.S Food and Drug Administration, 2023). This ctDNA *EGFR* mutation testing leads to cost reductions and enables more effective treatment, resulting in a positive economic impact. Table 1 shows other current ctDNA tests approved for application in the clinical management of different cancer types.

**TABLE 1:** FDA approved tests for identifying mutations used in liquid biopsy.

Year	Name test	Technology	Company	Biomarker	Molecular alteration	Cancer
2016	cobas® EGFR Mutation Test v2	real-time PCR	Roche Molecular Systems, Inc	*EGFR*	42 *EGFR* mutations in exons 18, 19, 20, and 21	NSCLC
2019	Therascreen PIK3CA RGQ PCR Kit	real-time PCR	QIAGEN GmbH	*PIK3CA*	11 mutations in exons 7, 9, and 20	Breast Cancer
2020	FoundationOne® Liquid CDx	NGS	Foundation Medicine, Inc	*PIK3CA*	*PIK3CA* mutations C420R, E542K, E545A, E545D [1635G>T only], E545G, E545K, Q546E, Q546R; and H1047L, H1047R, and H1047Y	Breast Cancer
				*BRCA1, BRCA2*, *ATM*.	*BRCA1, BRCA2, ATM* alterations	Prostate Cancer
				*BRCA1, BRCA2*	*BRCA1, BRCA2* alterations	Ovarian Cancer
				*MET, EGFR, ALK.*	*ALK, EGFR, MET*	NSCLC
2022	Agilent Resolution ctDx FIRST assay	NGS	Resolution Bioscience, Inc	*KRAS*	*KRAS* G12C	NSCLC
				*EGFR*	Single nucleotide variants (SNVs) and deletions	
2023	Guardant 360 CDx	NGS	Guardant Health	*ERS1*	*ESR1* missense mutations between codons 310–547	Breast Cancer
2023	FoundationOne® Liquid CDx	NGS	Foundation Medicine, Inc	*BRAF*	*BRAF V600E* alteration	Colorectal Cancer

Adapted table of U.S Food and Drug Administrations https://www.fda.gov/medical-devices/in-vitro-diagnostics/list-cleared-or-approved-companion-diagnostic-devices-in-vitro-and-imaging-tools and https://www.accessdata.fda.gov/cdrh_docs/pdf19/P190032S010A.pdf

PCR, polymerase chain reaction; NGS, Next-Generation Sequencing; Non-Small Cell Lung Cancer.

Advances in next-generation sequencing (NGS) technology and a large demand for ctDNA mutation analysis to support clinical studies have facilitated the emergence of sequencing assays covering cancer-related genes (Yu et al., 2022). Because it is rare, detection of mutations in ctDNA can be challenging, even with the increased feasibility of its analysis through NGS, which can present error rates of 0.1%–1% depending on the platform used (Glenn, 2011).

**FIGURE 2:**
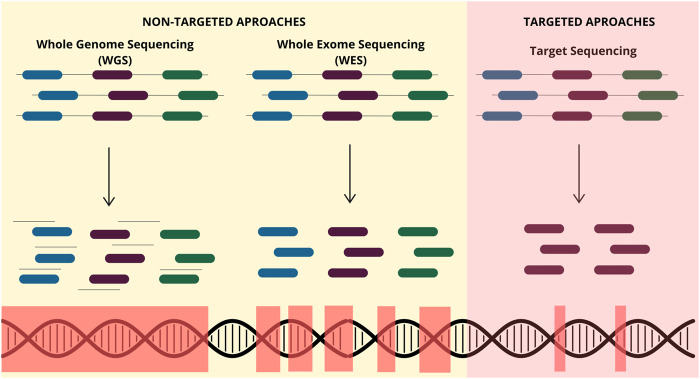
Different NGS-based approaches available for ctDNA analysis. The non-targeted approach includes whole-genome sequencing (WGS), which captures the entire genome from a biological sample, including coding and noncoding regions. Additionally, whole-exome sequencing (WES) captures only coding regions. Contrastly, targeted techniques capture only the molecular alterations of genes of interest that are previously known.

Currently, sequencing technologies have two distinct approaches with different methods and applications. The non-targeted sequencing often provides an overview of the entire genome and captures coding and non-coding regions. Also, it enables new genetic discovery without previous knowledge (Bagger et al., 2024). Conversely, targeted sequencing focuses on specific genes or regions of interest previously known, which participate in biological processes and diseases (Figure 2) (Singh, 2022).

Recently, long-read sequencers, known as third-generation sequencing (TGS), have emerged to surpass NGS technologies. This approach allows the reading of single DNA molecules in real time without the need for prior PCR amplification steps, offering high precision and speed. Furthermore, TGS is capable of detecting epigenetic modifications, and its rapid results make it attractive for disease diagnosis, particularly in precision oncology (Ling et al., 2023; Scarano et al., 2024).

In the present study, we described NGS and TGS approaches and discussed standardized methodologies and challenges for the identification of ctDNA mutation. Additionally, we explore cost-effectiveness, specificity, clinical utility, and bioinformatic implications for optimal NGS application in ctDNA analysis from cancer patients.

## Next-generation sequencing

The NGS technology has revolutionized the field of genomics by enabling rapid and affordable large-scale DNA and RNA sequencing. This methodology is based on analyzing several millions of short DNA fragments in parallel, followed by either sequence alignment to a reference genome or *de novo* sequence assembly (Lin et al., 2021). Therefore, this technology can be useful for real-time monitoring of tumor progression through detection with high accuracy of genetic status from primary and metastatic tumors (Hess et al., 2020).

Usually, library preparation is a critical step that precedes sequencing and varies according to study type and available financial resources. This process consists of ensuring genetic material is appropriate to be sequenced by high-throughput sequencing platforms and may include separation of large fragments, recovery of small fragments through probes, repair of DNA ends, connector connection, and addition of a special connector from the sequencing kit (Liang et al., 2020; Bohers et al., 2021). A technological advance within library preparation is the use of molecular barcoding by inserting random sequences prior to PCR amplification to obtain counts of original DNA molecules without unbiased results and with increased sensitivity (Bohers et al., 2021; Szadkowska et al., 2022).

In ctDNA, the identification of mutations is challenging due to its representation of a small fraction of cfDNA and the need for high levels of plasma DNA for analysis (Dang and Park, 2022). However, the various NGS tools offer potential applicability, specificity, sensitivity and low input, making them invaluable in ctDNA research (Elazezy and Joosse, 2018). This includes non-targeted (Diefenbach et al., 2019; Ganesamoorthy et al., 2022) and targeted approaches (Phallen et al., 2017; Elazezy and Joosse, 2018; Gale et al., 2018; Peng et al., 2019; Zhao et al., 2020; Kato et al., 2021; Hallermayr et al., 2022) (Table 2).

**TABLE 2:** Sequencing NGS- and TGS-bated methods used for ctDNA analysis.

Technology		Methods	Sensitivity (%)	Specificity (%)	Input (ng)	Applications	Alteration	Reference
NGS	Non targeted	WGS	5–10	99.85	1–30	Cancer localization and origin, early detection (early and late stage), for research us	Structural and non-coding variations: genome-wide copy number aberrations, methylation profiles and fragmentation patterns	(Ganesamoorthy et al., 2022)
		WES	5	96	5	Cancer detection, monitoring of resistant clones in metastasis, for research use	Exploring unknown mutations	Diefenbach et al. (2019)
	Targeted	Safe-SeqS/UMI-based	0.01–0.05	98.9	3	Cancer detection and monitoring, classification, targetable alterations, for research use	Known point mutation and number copy variation	(Elazezy and Joosse, 2018)
		Tam-Seq	2	99.9997	0.9–20	Cancer detection and monitoring, classification, targetable alterations, for research use	Known point mutation	(Gale et al., 2018)
		CancerSEEK	69–98	99	0.11–119	Early cancer detection	Mutations nonsense, insertions or deletions, synonymous mutations and intronic mutations	Cohen et al. (2018)
		eTam-Seq	0.2	99.9997	6.6–53	Cancer detection and monitoring, classification, targetable alterations, for research use	Low frequency mutations, short (indels)	(Gale et al., 2018)
		CAPP-SEQ	0.02	99.99	32	Molecular Profiling, Treatment Monitoring, ctDNA MRD	Known point mutation, number copy variation and rearrangements	(Kato et al., 2021)
		Ig-HTS	10–6	98.3	500	Minimal residual disease in hematologic malignancy and cancer monitoring	Not mentioned	Rezazadeh et al. (2024)
NGS	Targeted	TEC-Seq	0.05–0.01	99.99	2.9–49.5	Molecular Profiling, Treatment Monitoring, ctDNA MRD	Point mutations, small insertions, and deletions	Phallen et al. (2017)
		Single primer extension (SPE)	0.05–1	94	1–50	Cancer detection and monitoring, classification, targetable alterations, for research use	Point mutations	(Zhao et al., 2020)
		SPE-duplex UMI	0.1–0.2	95	40	Cancer detection and monitoring, classification, targetable alterations, for research use	Single-nucleotide variant and Indel mutations	(Peng et al., 2019)
		Duplex Sequencing	0.001–0.1	96.91	64	Cancer detection and monitoring, classification, targetable alterations, for research use	Known and unknown mutations, indels, CNV, chromosomal rearrangements (capture)	(Hallermayr et al., 2022)
TGS	Single Molecular Real-time		Not mentioned	Not mentioned	Not mentioned	Reading of repetitive elements and allele phasing in long fragments	Not mentioned	Choy et al. (2022)
	Nanopore	CyclomicsSeq	Not mentioned	Not mentioned	1500	Real-time monitoring of tumors	Nonsense mutation, missense and deletion	(Marcozzi et al., 2021)

WGS, Whole-genome sequencing; WES., Whole-exome sequencing; Safe-SeqS, Safe-Sequencing System; UMI, unique molecular identifier; Tam-Seq, Tagged-amplicon deep sequencing; eTam-seq, enhanced Tam-Seq; CAPP-Seq, Cancer Personalized Profiling by Deep Sequencing; TEC-Seq, Targeted error correction sequencing; Ig-HTS, Immunoglobulin high-throughput sequencing; SPE, single primer extension.

## Non-targeted NGS technologies

In the realm of non-targeted sequencing, the focus broadens to include the entire genome or exome using methods such as whole-genome sequencing (WGS) and whole-exome sequencing (WES), allowing for the simultaneous identification of multiple mutations (Elazezy and Joosse, 2018; Chen and Zhao, 2019; Esteva-Socias et al., 2020). In ctDNA analysis, these methodologies can be applied to discover new molecular alterations, recognize new drug targets, and screen for drug resistance clones (Bohers et al., 2021).

In particular, WGS technologies are better suited to identifying structural and non-coding variations in ctDNA, composing a potential promise for the diagnosis of rare diseases (Bos et al., 2020; Marshall et al., 2020; Sun et al., 2021; Ibañez et al., 2022). The goal of the technique is to detect mutations, chromosomal alterations, genetic rearrangements, and somatic copy number alterations (Daya and Mahfouz, 2018).

According to Zviran et al. (2020) the WGS approach allowed dynamic tracking of tumor burden and detection of single nucleotide variations in postoperative residual disease in colorectal cancer with sensitivity ±SE = 90% ± 0.069%, specificity ±SE = 98% ± 0.006% (AUC ±SE = 0.97 ± 0.025). In addition, showed an association with shorter recurrence-free survival for 36.8% (7/19) of post-operative ctDNA-positive patients *P* = 0.03.

Recently, a study used ultra-low-pass whole-genome sequencing (ULP-WGS), an emergent tool for ctDNA analysis in hepatocellular carcinoma (HC) patients. This technique is cheaper compared to WGS and has a total ctDNA input of 2.5 ng but a very low coverage (<0.05), which can leave gaps in the sequencing. The results showed that 30.1% (22/73) of HC patients had detectable ctDNA levels. Furthermore, a pattern of chromosomal changes was found, such as the loss of 5q (36.3%) and 16q (40.9%) with an association with positive ctDNA as a predictor of worse prognosis and a biomarker of tumor aggressiveness (Sogbe et al., 2024).

In contrast, WES is a limited method only for coding regions (Sabatier et al., 2022). It is generally used to detect genetic variants that are associated with diseases and detect mutations (Glotov et al., 2023). In a comparative study, WES was applied to paired ctDNA and tumor biopsy in 15 patients for breast cancer, sarcoma, gastrointestinal cancer and melanoma. It was observed that the ctDNA fraction <16.4% is insufficient for detecting tumor-specific variants with a median number of 3 variants, in contrast, a value >30% of ctDNA fraction detected 95 non-synonymous variants. Furthermore, the results showed that ctDNA captures tumor heterogeneity by sharing 22 variants between melanoma (primary tumor) and liver (metastatic) and 12 additional variants that are unique to a tumor site, as well as being able to identify more frequently mutated genes concordant between WES ctDNA and tissue for breast cancer such as *ESR1, KRAS, PIK3CA, PIK3R1, FAT1* and *MED12*, for gastrointestinal cancer *APC, CASP8, GRIN2A, MYH9, TP53, ASXL1, CDH11* and *KRAS*; and melanoma *PSIP1, RSPO2* and *SF3B1* (Leenanitikul et al., 2023).

Nevertheless, it is adequate to detect mutation in patients with advanced tumors and increased ctDNA fractions (Bohers et al., 2021). A study by Diefenbach et al., 2019 showed that ctDNA WES can be used to profile mutations and capture clinically relevant alterations in metastatic melanoma, such as *BRAF* and *NRAS* melanoma driver gene mutations in 6/10 patients when applying a mutant allele frequency (MAF) cutoff of at least 10%.

Notably, WES presents a cost-effective approach compared to WGS by exclusively scrutinizing exons. However, both WGS and WES demand substantial DNA input to ensure the acquisition of high-quality data for the sequencing process and high-throughput. Therefore, these techniques are expensive, which makes their clinical application challenging. Additionally, these methods exhibit limited sensitivity, rendering them less suitable for early-stage cancer detection (Ganesamoorthy et al., 2022).

## Targeted NGS-based methods

The targeted strategies allow the detection of single or few tumor-specific mutations in ctDNA through pre-selected panels previously described, such as *BRAF, KRAS, TP53, PIK3CA, APC* and *EGFR* (Elazezy and Joosse, 2018; Mallampati et al., 2019; Liu et al., 2020; Kato et al., 2021; Jiménez-Rodríguez et al., 2022). These techniques could be useful in clinical management for monitoring MRD, early detection of relapse or screening for resistant mutations (Bohers et al., 2021; Lin et al., 2021; Sanz-Garcia et al., 2022).

Generally, customized panels are constructed based on mutations captured during tissue sequencing and applied to detect tumor-specific mutations in plasma (Sanz-Garcia et al., 2022). In addition, laboratories have no standardization in the clinical implementation of NGS panel design. It is widespread to use pre-designed panels from suppliers or to create your panels. However, developing a targeted panel from scratch is challenging, as investments in operational infrastructure and bioinformatics are required (Shi et al., 2022).

### Amplicon

**FIGURE 3:**
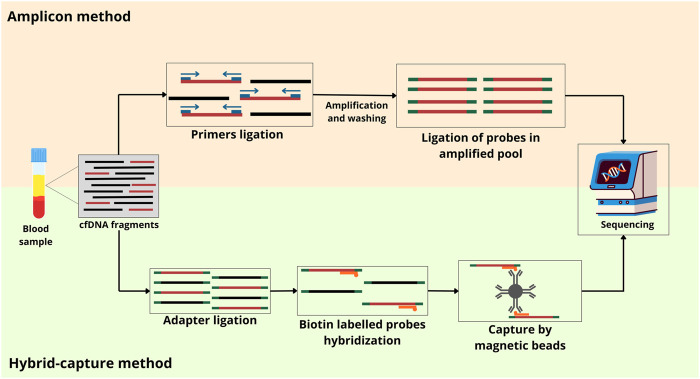
Two NGS-based targeted approaches for ctDNA analysis. The amplicon approach is based on the PCR method, which amplifies specific regions of the genome. The hybrid-capture approach uses probes to capture and enrich specific genomic regions of interest before sequencing. cfDNA, cell-free DNA.

Target NGS technologies require enrichment by amplicon or hybrid-capture (Figure 3) (Lin et al., 2021; Sanz-Garcia et al., 2022). Amplicon sequencing, a targeted NGS method able to analyze genetic variation in specific genomic regions, consists of a multiplex PCR-based method that uses oligonucleotides to target and capture regions of interest. PCR is used to create DNA sequences known as amplicons, which can be multiplexed by adding a barcode or index to the samples for identification. Before, the samples must be transferred into libraries by adding adapters and enriching targets using PCR amplification. The adapters allow the formation of indexed amplicons and their adherence to the flow cell for sequencing (Hung et al., 2018). Currently, some amplicon-based methods are described in the literature.

**FIGURE 4:**
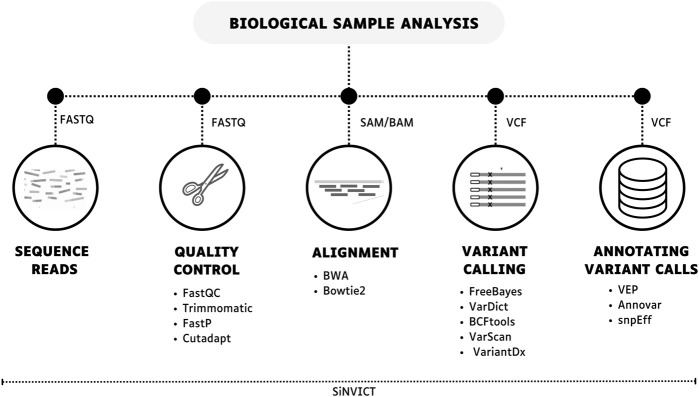
Bioinformatics workflow for data-seq for ctDNA evaluation. This process generally includes obtaining sequence reads, performing quality control, genomic alignment, variant calling, and annotating variant calls. Multiple tools are available for each step, or a single tool can be used to complete all the steps (SiNVICT).

### Safe-sequencing system (Safe-SeqS)

Safe-SeqS is an amplicon method that uses DNA molecular barcodes to increase sequencing sensitivity before PCR and uses the unique identifier (UID), which allows fragments with the same UID to be considered mutants if more than 95% have the same mutation. Barcode error correction increases sensitivity to 0.05% and identifies rare mutations (Tuaeva et al., 2019; Bohers et al., 2021). Tie et al. (2021) designed Safe-SeqS to evaluate a previously detected mutation with a higher allele frequency in 54 patients with resectable colorectal liver metastases (CRLM) and evaluated the prognostic impact of postoperative ctDNA in patients with CRLM. As a result, ctDNA was most detectable in patients at baseline (T0) 85% (46/54) with a median MAF for positive ctDNA of 1.86% (IQR, 0.44%–8.2%) and in patients after surgery (TP) 24% (12/49) 0.09% (IQR, 0.02%–1.3%).

Nowadays, Safe-seqS is recognized as Unique Molecular Identifier (UMI)-based sequencing and highlights in new nomenclature the use of unique molecular identifiers (UMIs) to track and correct errors during the process, with greater accuracy in the detection of rare mutations and in the quantification of nucleic acids (Salk et al., 2018). UMI-based sequencing technology was used to investigate somatic mutations in ctDNA of patients with lung squamous cell carcinoma (LUSC), which were detected in 80.8% (20/26) of patients and mutations with maximum allele fraction (maxAF) > 5% compared to maxAF ≤5% (*P* = 0.020) reflected shorter overall survival. The most frequently mutated gene was *TP53* with 73.0% (19/26), and the classic lung cancer driver mutations, *PIK3CA* (n = 3), *EGFR* amplification (n = 2), *EGFR* exon 19 deletion (n = 1), *KRAS* Q61R (n = 1), and *MET* amplification (n = 1) were detected (Liu et al., 2020).

### Tagged-amplicon deep sequencing (Tam-seq)

Tam-seq uses an enrichment matrix with primers and barcodes in the construction of an amplicon library, which goes through steps of targeted pre-amplification and selective amplification with single-plex reactions, as well as PCR is performed for the addition of adapters and barcodes for sample identification (Zhao et al.,2020). This technique showed high sensitivity 0.01%–2.0% and specificity >97% to detect mutations in circulating DNA, as a ctDNA analysis method that allows for an ultra-low detection limit and broad patient coverage, as well as showing digital PCR-like sensitivity for hotspot alleles and can simultaneously interrogate thousands of additional genomic positions without your sensitivity or specificity are affected (Noguchi et al., 2020). The technique requires knowledge of recurrent cancer mutations available in databases and uses a selector (biotinylated oligonucleotide probes) to target large segments of the studied regions (Bohers et al., 2021).

In 2018, Gale et al. described enhanced Tam-Seq (eTam-Seq), which consists of an expanded assay to target hotspots and entire coding regions of 35 genes for common cancer types, based on a primer design that allows amplification of highly fragmented DNA and in library preparation does not use microfluidics. This technique aims to identify single nucleotide variants (SNVs) and short insertions/deletions (indels) and identify copy number variants (CNVs). The validation test results of this tool indicated high specificity 99.9997% (95% (CI): 99.9989%–99.9999% by base specificity) and sensitivity 100% (90% (CI): 99.01%–100%) in low input samples at 2%–2.5% AF, 99.17% (90% CI: 97.40%–99.85%) in medium input samples at 1%–1.3% AF and 95.45% (90% CI: 93.09%–97.18%) in high input samples at 0.25%–0.33% AF (Gale et al., 2018).

On the other hand, the hybrid-capture, also known as hybridization-based sequencing, is based on using long, biotinylated probes or baits complementary to the region of interest. This method involved the fragmentation of physical or enzymatic DNA followed by enzymatic repair of the ends of the molecules and ligation of platform-specific adapters. These adapters usually contain index bases that comprise a sequence that is unique to the sample or the barcode of the sample (Bohers et al., 2021). Unlike amplicon sequencing, this method does not require PCR primer design. Thus, it is less likely to miss mutations and is said to be better at performing in terms of sequence complexity. The capacity of this method for mutation detection makes it best suited to cancer research. Moreover, its sequence complexity and scalability make it good for WES (Wu et al., 2022).

### Hybrid capture

When choosing panels in the hybridization method, cfDNA fragmentation must be taken into account, as it may result in heterogeneous coverage between target exons (Lin et al., 2021; Shen et al., 2021). This enrichment step prevents loss of the variant of interest if they are on the edges of the fragments because the probe binding to the target region is sufficient to capture the variant. However, the fragments may not amplify because they do not have a binding sequence with the primers during NGS library preparation (Mallampati et al., 2019). Several hybrid capture-based technologies have been described.

### Cancer personalized profiling by deep sequencing (CAPP-Seq)

CAPP-Seq developed the ability to simultaneously detect several types of changes: SNVs, rearrangements, insertions/deletions, and copy number changes (Elazezy and Joosse, 2018). Additionally, CAPP-Seq has been enhanced with Integrated Digital Error Suppression (iDES), combining CAPP-Seq with duplex barcode sequencing technology and a computational algorithm that removes stereotyped errors associated with the CAPP-Seq hybridization step (Peng et al., 2019). According to Kato et al. (2021), CAPP-SEq applied to ctDNA mutation analysis allowed the identification of mechanisms of resistance to osimertinib in *EGFR* T790M-positive NSCLC patients. In addition, the assay also detected *EGFR*-activating mutation in 70% (14/20) of patients, and these results were associated with a larger tumor volume through the sum analysis of the largest diameters of the target lesions (*P* = 0.04). In addition, for patients with *EGFR* activating mutation, mutations were observed in the genes *PIK3CA* (3/14) 21%, *KRAS* (2/14) (14%) and or *BRAF* (3/14) 21% and copy number gain alterations for *EGFR* (9/14) 64%, *ERBB2* (4/14) 29% or *MET* (4/14) 29%. Additionally, the identified alterations were more common in patients with innate resistance 8 (57%) compared to patients with acquired resistance 6 (43%) (Kato et al., 2021).

### Others technologies

Some approaches described use different combinations of technologies to optimize results. Some methods do not apply to the amplicon enrichment or hybrid capture standards.

### Immunoglobulin high-throughput sequencing (Ig-HTS)

Ig-HTS is an ultra-deep genomic DNA sequencing method developed for minimal residual disease in hematologic malignancy that uses multiplex PCR arrays to identify a tumor-specific clonotype from rearranged gene regions of IgH, IgK, and IgL receptors. This technology enables cancer monitoring through quantifying ctDNA with a sensitivity of 10%–6% (Bohers et al., 2021). In 2022, Rezazedeh et al. demonstrated that Ig-HTS as a Food and Drug Administration-proven tool clonoSEQ (Adaptive Biotechnologies) allows the minimization of surveillance imaging in patients with B-cell lymphomas from ctDNA analysis, in which the result of the MRD assay was predictive of relapse before imaging in 92% of patients (11/12) (Rezazadeh et al., 2024).

### Targeted error correction sequencing (TEC-Seq)

TEC-Seq is a method that combines targeted sequencing and error correction approaches, which has a sensitivity of 94.7% and is capable of detecting mutations in early-stage solid cancers, as well as being a method capable of identifying true mutations and false-positive variants (Phallen et al., 2017; Bohers et al., 2021). Serrano et al. employed TEC-Seq for serial monitoring of ctDNA from patients with gastrointestinal stromal tumors to evaluate the combination of sunitinib and regorafenib as a new add-on drug treatment regimen. In this study, somatic mutations, point mutations, small insertions, and deletions were analyzed. This approach resulted in primary mutations in 89% (8/9) and secondary mutations in 78% (7/9) of patients (Serrano et al., 2019).

### Single primer extension (SPE)

SPE is a method developed by QIAGEN that redefines amplicon enrichment and sequencing (QIAseq SPE technology for Illumina: Redefining amplicon sequencing - QIAGEN, 2018). The method is based on the extension of a single gene-specific primer by DNA polymerase to amplify each genomic region with uniform coverage, allowing the detection of single nucleotide polymorphisms (SNPs) and specific mutations with high accuracy. Initially, the primer is hybridized to the DNA template strand in the target region, where there are subsequent adapter ligation repair steps. Then, the primer is extended from the 3′ end, and each genomic region is targeted by only one region-specific primer plus a universal adapter primer that binds to sequences introduced through adapters. These adapters are linked to primers and a molecular barcoding technology used to uniquely tag each molecule in the sample library, Unique Molecular Index (UMI), with a sensitivity of 0.5%–1% (Bentley et al., 2008; Peng et al., 2019; Zhao et al., 2020). In SPE, the use of UMI reduces amplification errors and increases the sensitivity of variant detection, which provides error correction and higher accuracy during sequencing. Additionally, SPE can be enhanced through duplex UMI adapters (duplex SP-UMI), multiplex PCR-based enrichment and sequencing, which increases sensitivity to 0.1%–0.2% (Peng et al., 2019).

Recently, this technology was used by Jiménez-Rodríguez et al. (2022) for the analysis of ctDNA from BC patients and a sequencing panel composed of exonic regions of 33 genes in 75 plasma samples was developed. As a result of the study, 21.31% (13/61) of tumor mutations were found in both plasma and corresponding tumors, and the most frequently mutated genes were *TP53* (53.84%) and *PIK3CA* (23.07%). In addition, it presented a sensitivity of 0.03% and a specificity of 86.36%.

### Duplex sequencing

Duplex sequencing is a method that aims to achieve accuracy and reduce sequencing errors based on double-strand consensus analysis. This technique begins with the fragmentation of DNA into smaller pieces and the addition of specific adapters. The fragmented DNA is encapsulated in emulsion drops where PCR amplification occurs, generating single-strand readings. The single strands are paired to form duplex readings. The analysis of the two strands is compared to eliminate random errors that can be identified by the lack of correspondence between the single-strand readings (Mallampati et al., 2019; Bohers et al., 2021; Shields et al., 2022). This approach was demonstrated by Mallampati et al. (2019) to monitor disease progression in patients with stage IV colorectal cancer. In this research, a CRC23 panel with 78.81 kb was created involving 85% of mutated targets and exon regions for the *TP53, APC, KRAS, NRAS, BRAF, PIK3CA* and *ERBB2* genes and hotspot coding exons of 16 other genes. Furthermore, a detection limit of 0.3% of variant frequency was observed, as well as diagnostic accuracy of 96.15% (95% CI, 94.28%–97.55%), sensitivity of 87.23% (95% CI, 74.26%–95.17%) and specificity of 96.91% (95% CI, 95.11%–98.19%).

Although the targeted strategy makes cancer monitoring extremely sensitive, these approaches require prior genetic knowledge of the tumor. This may not be useful in characterizing new molecular alterations that occur during tumor treatment (Elazezy and Joosse, 2018; Sanz-Garcia et al., 2022).

## Third generation of sequencing

Additionally to NGS, the advent of the third generation of sequencing (TGS) has provided new features and capabilities for real-time reading, long-fragment reading, portability, and ease of use which are fundamental to understanding cancer genetics, and currently PacBio Sequencing (Menlo Park, CA, United States) and Oxford Nanopore Technologies (ONT, Oxford, United Kingdom) are the two TGS technology platforms (Amarasinghe et al., 2020; Scarano et al., 2024).

Single Molecular Real-Time (SMRT) (Pacific Biosciences, California) is a method based on reading made on SMRT chips which is composed of metal film containing zero-mode waveguides (ZMW) which are special nanophotonic visualization chambers. Inside chambers in the flow cell are ZMW that capture signals from phospholinked dNTP labeled with fluorophores which are incorporated by DNA polymerase and released fluorescence pulse that is identified by laser at a specific wavelength in real time (Treffer and Deckert, 2010). This SMRT technology enables the reading of repetitive elements and allele phasing in long fragments (Ardui et al., 2018). In the analysis of ctDNA, SMRT sequencing was used to evaluate long DNA properties and methylation patterns, since analyses usually focus on short fragments. The assay results showed the detection of fragments up to 13.6 kb in length in samples from 13 patients with hepatocellular carcinoma. Additionally, it was observed that non-tumor cfDNA was generally longer than tumor cfDNA, in which plasma DNA molecules longer than 600 bp were 55.1% carrying mutant alleles and 64.8% wild-type, and molecules longer than 1 kb were 43.4% carrying mutant alleles and 56.4% wild-type. Furthermore, complete reads were performed in 85.79% (IQR: 83.11%–88.69%) of the fragments. Another important point to be analyzed was the detection of long cfDNA fragments containing a mutant allele, which can generate changes in cfDNA analyses for the inclusion of long molecules (Choy et al., 2022).

Furthermore, nanopore sequencing (Oxford Nanopore Technologies) is a technology that consists of real-time readings of changes in electrical current during the passage of the DNA molecule through a biosensor, which is composed of an electrically resistant membrane. The nanopores are arranged in the flow cell in micro-scaffolds and can be categorized as solid and biological. Each nanopore is an electrode connected to the channel inside the sensor chip where the electrical current is measured. When the electrical current is interrupted by the passage of a molecule, the so-called “squiggle” occurs and this information becomes corresponding to a specific nucleotide. This method has capacity for long-read sequencing, empowering the direct analysis of DNA or RNA fragments sans the prerequisite of prior amplification (Wang et al., 2021; Scarano et al., 2024). This TGS technology was employed to analyze genomic and fragmentomic data from liquid biopsies in 8 urine samples from bladder cancer patients and 22 plasma samples from lung cancer patients. ONT sequencing performed on the MinION showed structural properties of cfDNA and the ability to recover somatic copy number aberrations (SCNAs) in 24 h with a median of 800,183 reads and ∼0.1X coverage. Although cfDNA is described in the literature as short and fragmented molecules (167 bp), the results obtained from this research showed increased recovery of long cfDNA (>300 bp) in plasma from lung cancer patients, and compared to short-read sequencing (5.3%), ONT sequencing had 54.1% of fragments larger than 300 bp (van der Pol et al., 2023).

CyclomicsSeq is a technology based on the circularization and concatemerization of DNA molecules and an optimized DNA sequence in combination with Oxford Nanopore sequencing created for real-time monitoring of tumors based on the analysis of ctDNA levels. The protocol of this technology uses amplicons and is divided into four steps, which involve the circularization of the insert and backbone (DNA adapter), rolling circle amplification (RCA), long-read sequencing and data processing. The detection of ctDNA through this technology allows the identification of mutations based on somatic variants. Real-time monitoring can be done by identifying mutations in the *TP53* gene, in which a *TP53* mutation was observed in a trial with patients with head and neck squamous cell cancer negative for the human papillomavirus (HPV) at a frequency of 0.02%. During the trial, the single nucleotide error false positive rate (snFP rate) was also analyzed, which had a median <6, 10^−4^ in all *TP53* exons to evaluate the use of CyclomicsSeq for mutation detection in liquid biopsy (Marcozzi et al., 2021).

Although TGS can generate long reads and detect complex structural variants, its use in ctDNA analysis still has challenges. ctDNA fragments are rare in cfDNA, and reads of long fragments can induce the appearance of false base substitution mutations and indels (Ardui et al., 2018; Marcozzi et al., 2021; Scarano et al., 2024). These errors can make it difficult to accurately detect relevant mutations that could interfere with the clinical management of cancer patients.

## Sequencing data analysis

Data sequencing analysis is a critical process for ctDNA evaluation and consists of three main steps: quality analysis, alignment, and variant calling (Figure 4) (Wadapurkar and Vyas, 2018). Firstly, quality control of the reads is crucial for the bioinformatics analysis since high throughput NGS generates a massive volume of data and improves confidence in the data. In general, programs like FastQC provide a comprehensive per-base analysis, ensuring that the sequence is accurate and not compromised by issues generated during the sequencing run (Andrews, 2010; Trivedi et al., 2014; Mahamdallie et al., 2018). Moreover, reads can be contaminated by other sequences, such as primers or adapters in library preparation. Thus, several tools may be used to remove low-quality bases and sequences from adapters, such as Cutadapt, FastP, and Trimmomatic (Bolger et al., 2014; Chen et al., 2018; Martins et al., 2021).

Based on the provenance of the data and the size of the fragments, several aligners can be useful for ctDNA, including BWA and Bowtie2 (Li and Durbin, 2009; Langmead and Salzberg, 2012). In target sequencing, the alignment process consists of comparing the generated sequences to verify the degree of similarity using a reference genome or a customized file containing only the regions of interest of the study as a parameter. Moreover, it is worth noting that the version of the genome used during the analysis should be the same in order to avoid later disagreements (Reinert et al., 2015; Dilliott et al., 2018; Kang et al., 2020).

The last step seeks the identification of variants that differ from the reference used, typically FreeBayes, VarScan, BCFtools, VarDict and VariantDx are among the tools used to find SNPS, indels during the calling process in ctDNA analysis (Liu et al., 2013; Kang et al., 2020). Finally, the variants found go through the annotation process, which is querying existing databases. The VarDict is an ultra-sensitive variant caller pipeline that has already been used for the identification of ctDNA variants in cancer samples (Lai et al., 2016; Leal et al., 2020).

A sufficient number of reads is extremely important for correct mapping, identifying genetic alterations, and ruling out putative execution errors, especially data from devices that show errors in base changes. Targeted sequencing provides just that, contributing to the identification of variants at low abundance, which is characteristic of ctDNA. Therefore, high coverages (>30,000×) are expected in this type of experiment.

In addition, variant detection in ctDNA samples can be challenging due to the low frequency of total cfDNA and PCR artifacts in library preparation. Thus, Kockan et al. (2017) introduced SiNVICT, which consists of a tool for the detection of SNVs and short indels in ctDNA at very low variant allele percentages with high accuracy and sensitivity. This approach includes pre-processing, SNV/indel calling, and post-processing steps. SiNVICT also allows for analyzing samples collected at different time points and evaluating the temporal clonal evolution of tumors, which could be useful for the detection of resistance mutations and therapy selection (Kockan et al., 2017).

## Conclusion and future perspectives

Currently, ctDNA analysis represents a crucial approach to guide cancer diagnosis, management and monitoring, but the clinical implementation of ctDNA is still limited (Oliveira et al., 2020). NGS has shown great potential for advancing clinical practices through the development of a diverse panel for identifying ctDNA mutations in different cancer types, but finding the optimal approach remains a challenge (Table 3). Studies based on non-targeted NGS have the highest cost but are necessary for the construction of mutational panels, especially in cases of tumors lacking biomarkers (Hess et al., 2020; Christodoulou et al., 2023). With these studies, it is expected that new techniques will be developed to detect ctDNA mutations even at low frequencies in the bloodstream.

**TABLE 3:** Sequencing technologies are available for ctDNA analysis, as well as its principles, advantages, and disadvantages.

Sequencing technology	Classification	Method	Principle	Advantages	Disadvantages
NGS	Non-targeted	WGS	Determining the complete DNA sequence from a genome captures exons (coding) and introns (non-coding) regions, providing a comprehensive view of the genetic information	Provides a genome-wide view, capturing all genetic variations without requiring prior knowledge of regions of interest	Presents high cost and generates large amounts of data, requiring substantial computational resources for analysis
		WES	Performs only sequencing of the coding regions of the genome	It is cost-effective and efficient in identifying clinically relevant mutations	Does not provide information on non-coding regions and it also requires comprehensive bioinformatics tools for analysis
	Targeted	Amplicon	Analyze genetic sequences by amplifying specific regions of the genome before sequencing	Exhibits high sensitivity, is customizable according to the needs of the study, has high performance, and has a shorter response time	Only provides information about the selected regions; the design of primers for regions with high genetic variability can be complex, and errors arising from the amplification steps can lead to false-positive results
		Hybrid-capture	Uses biotinylated oligonucleotide probes to hybridize and enrich the regions of interest before sequencing	It has high coverage and specificity, can be targeted to various genomic regions, and has no amplification bias	The workflow is more complex, expensive, and time-consuming due to the steps in the protocol. Errors in hybridization can lead to inadequate capture and false results
TGS		SMRT	Based on SMRT (Single Molecule, Real-Time) chips, fluorophore-labeled nucleotides are added to DNA polymerase, and when incorporated into the DNA strand, fluorescent light is recorded at a specific wavelength	Long DNA sequence reads allow identification of structural rearrangements and mutations that may be difficult to detect with short-read methods	Limitation on coverage and processing time
	Nanopore	CyclomicsSeq	Performs amplification and repeated cyclic reading of circular DNA molecules to achieve accurate detection of low-frequency variants	Presents high precision and sensitivity for detecting low-frequency mutations, and random errors are reduced due to the cyclic reading of the fragments	It has a high cost and technical complexity for its execution, in addition to having a lower yield compared to NGS and requiring sophisticated bioinformatics tools to analyze the results

NGS, Next-Generation Sequencing; TGS, third generation sequencing; WGS, Whole-Genome Sequencing; WES, Whole-Exome Sequencing; SMRT, Single Molecular Real-time.

One of the tests approved by the FDA based on NGS panels most used in clinical oncology practice is still Foundation One® Liquid Cdx, used with both tissue biopsies and ctDNA in NSCLC, breast, prostate, ovarian, and colorectal cancer (Newman et al., 2016; Shahnoor et al., 2023). This test allows comprehensive genomic profiling that guides more effective therapy and predicts patient prognosis (Woodhouse et al., 2020).

Another technology that is quite promising for application in clinical practice is CancerSEEK is an amplicon-based method that uses multiplex PCR in the enrichment step and was developed in 2018 as a blood test for early cancer detection through quantifying the levels of circulating proteins and cfDNA (Cohen et al., 2018; Duffy et al., 2021; Dao et al., 2023).

CancerSEEK is capable of detecting 8 types of non-metastatic cancer (ovarian, liver, stomach, pancreas, esophagus, colorectal, lung or breast) through the construction of a panel for 16 genes (*NRAS, CTNNB1*, *PIK3CA, FBXW7, APC, EGFR, BRAF, CDKN2A, PTEN, FGFR2, HRAS, KRAS, AKT1, TP53, PPP2R1A, GNAS*) composed of 61 amplifiers containing on average 33 base pairs each amplicon. This assay has shown results, after application in 1,005 patients, of sensitivities of 69%–98% for 5 types of cancer (ovarian, liver, stomach, pancreas and esophagus) and specificity >99% in 0.86% (7/812) of healthy controls. In addition, it was observed that the maximum ctDNA detection capacity of the assay could vary according to the type of tumor (60% for liver cancer and 100% for ovarian cancer) and DNA concentrations in plasma ranged from 0.11 to 119 ng/mL. The test identified rare mutations: nonsense, insertions or deletions, canonical splice site mutations, synonymous mutations, except at exon ends and intronic mutations, except at splice sites. Regarding the reading model, CancerSEEK uses reference sequences and custom scripts in Python, SQL and C# (In Silico Solutions, Falls Church, VA) (Cohen et al., 2018).

Although the CancerSEEK test has been recognized as a Breakthrough Device by the U.S. Food and Drug Administration for the detection of genetic mutations and proteins associated with pancreatic and ovarian cancers, it still needs to be validated in large-scale screening studies for commercialization (Duffy et al., 2021).

Therefore, it is expected that more target NGS-based technologies will be developed to increase the sensitivity of ctDNA detection. Additionally, as NGS-based experimental designs become more affordable and popular, there is an escalating demand for software capable of collating, manipulating, and visually presenting quality control (QC) logs and reports, especially when dealing with a substantial number of samples. Also, multiple factors, including cost, yield, specificity, cancer type, disease stage, clinical application, and bioinformatics analysis need to be considered.
